# Modelling cultural selection on biological fitness to integrate social transmission and adaptive explanations for human behaviour

**DOI:** 10.1017/ehs.2020.12

**Published:** 2020-04-23

**Authors:** Alberto J. C. Micheletti

**Affiliations:** Institute for Advanced Study in Toulouse, Université Toulouse 1 Capitole, 1 esplanade de l'Université, 31080 Toulouse Cedex 06, France

**Keywords:** Cultural evolution, cultural relatedness, cultural selection, biological fitness, adaptation

## Abstract

One of the difficulties with cultural group selection theory highlighted in the review by Smith (2020, *Evol. Hum. Sci.*, 2, e7) is its inability to separate the evolutionary effects of selection of cultural traits based on biological fitness (Cultural Selection 1) from the effects of selection based on cultural fitness (Cultural Selection 2). Confusing these two processes can hinder the integration of adaptive explanations for human behaviour, which focus on biological fitness, and cultural evolution explanations, which often focus on social transmission. Recent empirical work is starting to bridge this gap, but progress in mathematical modelling has been considerably slower. Here, I suggest that modellers can contribute to achieving this integration by further developing models of Cultural Selection 1, where behaviours are influenced by culturally inherited traits selected on the basis of their effects on biological fitness. These models should build on existing social evolution theory methods and replace genetic relatedness with cultural relatedness, that is the probability that two individuals share a cultural variant.

## Graphical abstract

**Figure:**
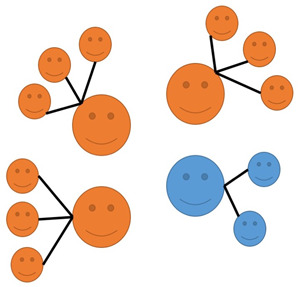

**Media summary:** Cultural evolution theory has shown that behaviours that are learnt socially or culturally – rather than inherited genetically – can evolve in a Darwinian fashion. Two selective processes can potentially lead to a culturally transmitted behaviour becoming widespread. In Cultural Selection 1, a behaviour is selected because it results in an individual having more children. Instead, in Cultural Selection 2, it is selected because it results in more ‘cultural children’, that is more learners or apprentices. I argue that distinguishing between these two processes is crucial and that building mathematical models of Cultural Selection 1 can help the advancement of cultural evolution theory.

Smith (2020) provides an insightful and balanced review of cultural group selection (CGS) theory. While underlining the merits of the theory, he also highlights several difficulties with it, which mean that the explanation for human social evolution offered by CGS in its current form is not entirely satisfactory. One such difficulty is the theory's inability to clearly separate the effects of selection of cultural traits based on an individual's biological fitness (i.e. number of offspring) from the effects of selection on her cultural fitness (i.e. number of apprentices/learners) (Smith 2020, pp. 17–18,22). As these two processes – recently termed Cultural Selection 1 (CS1) and Cultural Selection 2 (CS2), respectively (Birch 2017) – can lead to different evolutionary outcomes, failing to distinguish between them can cause confusion (see Fig. 1 for an illustration).

**Figure 1.**
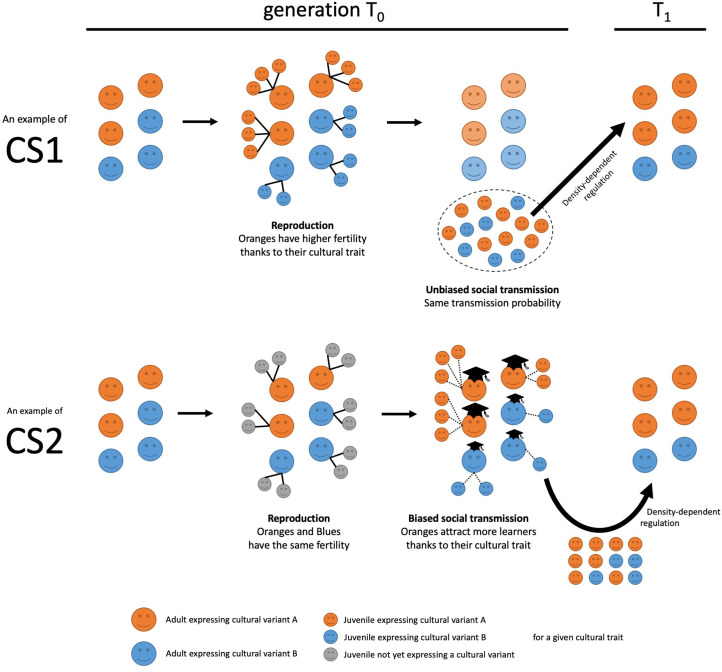
Cultural Selection 1 (CS1) and Cultural Selection 2 (CS2): an illustration. In both processes, orange icons represent individuals expressing cultural variant A, and blue icons represent individuals expressing a different cultural variant, B, for a given cultural trait (grey icons represent juveniles who do not have a cultural variant for the trait yet). In CS1: (1) adults reproduce asexually, with Oranges having higher fertility than Blues thanks to their cultural variant, and they transmit their cultural variant to their offspring; (2) unbiased horizontal transmission of cultural variants occurs between juveniles; (3) density-dependent regulation occurs, with a limited number of randomly chosen juveniles reaching adulthood. In CS2: (1) adults reproduce asexually, with Oranges and Blues having equal fertility, and their offspring not having either cultural variant for the trait at the start; (2) vertical and oblique transmission of cultural variants occurs, with juveniles learning a cultural variant from adults and with Oranges having a greater ability to attract learners than Blues thanks to their cultural variant; (3) density-dependent regulation occurs, with a limited number of randomly chosen juveniles reaching adulthood. In both cases, effects of random sampling are ignored to better illustrate the action of the two selective processes. Notice that these are just two examples (making specific assumptions about cultural transmission) and they are simply meant to illustrate how CS1 and CS2 can operate.

Moreover, I believe that lack of clarity concerning CS1 and CS2 – in the context of CGS and cultural evolution more in general – can hinder the integration of explanations based on social learning and adaptation to different ecologies. Recent empirical work has made some progress towards such an integration (Botero *et al.* 2014; Colleran 2016; Mattison *et al*. 2018). Here, I suggest that mathematical modelling of human social evolution can move in the same direction by further developing models of CS1. This should be done by building on existing social evolution theory methodologies and replacing genetic relatedness with cultural relatedness, i.e. the probability that individuals share a cultural variant (Allison 1992; Lehmann *et al*. 2008; Bell *et al.* 2009; Boyd *et al.* 2011; Birch 2017; Handley and Mathew 2020).

Human behaviours are influenced by both genetically inherited factors (genes) and culturally inherited factors, which may follow different evolutionary trajectories. Social evolution theory in biology has revealed that genetic natural selection tends to lead to adaptive behaviours that maximize an individual's inclusive fitness, i.e. her own reproduction and that of social partners, weighted by her genetic relatedness to them (Hamilton 1964; Grafen 2006; Gardner 2009; Lehmann and Rousset 2020). Genetic relatedness measures the probability that two individuals share a given gene, relative to the population average, and generally coincides with genealogical kinship (Frank 1998). Human behavioural ecology has applied inclusive fitness theory to the study of human behaviour with considerable success. However, this approach has tended to consider culture only as a proximate mechanism that does not to pose constraints on evolution (the ‘phenotypic gambit’; Nettle *et al.* 2013).

Yet should we expect the evolution of culturally inherited traits to lead to the same adaptive outcomes as the evolution of genetically inherited ones? To answer this question, it is crucial to distinguish between CS1 and CS2. In recent years, cultural selection on cultural fitness (CS2) has received considerable attention, with several suggestions that this process can lead to biologically maladaptive traits (e.g. Henrich 2004a; Richerson and Boyd 2005; Mesoudi *et al.* 2006; Tanaka *et al.* 2009). CGS has played an important role in these efforts, because selection of group-level cultural traits through between-group competition always involves CS2, but not always CS1, as Smith (2020, p. 18) clarifies.

On the other hand, CS1 has been relatively neglected. In this selective process, the currency is the same as in genetic evolution, but the transmission of the cultural variants determining the behaviour is potentially different, because they can be inherited vertically, obliquely (from a non-parental adult), horizontally (within a generation) or in a combination of these modes (Birch 2017). Thus, rather than genetic relatedness, we need to consider cultural relatedness, that is the probability that two individuals share a cultural variant (Allison 1992; Lehmann *et al*. 2008; Bell *et al.* 2009; Boyd *et al.* 2011; Birch 2017; Handley and Mathew 2020). For a given trait, cultural relatedness can in principle be higher, lower or equal to genetic relatedness. If horizontal and oblique transmission are unbiased, then the *qualitative* predictions of a CS1 model – for example, regarding the direction of sex differences in behaviour – would be the same as for a genetic model. However, predictions might differ *quantitatively* – i.e. the magnitude of those sex differences might differ – because cultural relatedness can be higher than genetic relatedness (Lehmann *et al*. 2008; Micheletti *et al.* 2020).

Some steps in the analysis of CS1 have been taken. Lehmann *et al.* (2008) and Lehmann and Feldman (2008) have shown that altruism can evolve more or less readily under CS1 than genetic selection depending on social learning modes (but see Boyd *et al.* 2011). In addition, Birch (2017) has suggested that CS1 might have driven the evolution of prosocial tendencies and has proposed an expanded definition of relatedness that captures horizontal transmission. Notwithstanding this work, CS1 is generally not being employed to model specific questions of human social evolution. To encourage this, methods to build CS1 models should be presented in an accessible format, which clarifies the key assumptions concisely and includes some worked examples (e.g. modelled after Taylor and Frank 1996). Moreover, more formal theory should be developed to analyse the effects of different forms of transmission (e.g. including non-vertical transmission, biased or unbiased; starting from Lehmann *et al.* 2008; Lehmann and Feldman 2008; Boyd *et al.* 2011; Birch 2017) and explore interaction with CS2 processes (starting from Henrich 2004b; El Mouden *et al*. 2014).

In conclusion, modelling CS1 – selection of cultural variants based on their effects on biological fitness – can act as a bridge between social learning explanations, which often focus on cultural transmission, and adaptive explanations, centred on inclusive fitness. Moreover, employing CS1 models, instead of genetic ones, would greatly facilitate interaction between empiricists and theoreticians, especially for behaviours that have a clear and strong culturally inherited component (e.g. post-marital residence). Importantly, such efforts would not necessitate the development of models *ex novo*. Instead, they should build upon the already advanced mathematical machinery of genetic social evolution theory (Frank 1998; Rousset 2004). Thus, this new class of models could be intended as a ‘cultural’ expansion of the Hamilton's rule organising framework (Birch 2017), creating a natural bridge between inclusive fitness and cultural evolution approaches.
